# Genome-Wide Identification of the *BhKCS* Gene Family in Wax Gourd (*Benincasa hispida*) and Stress-Responsive Expression of *BhKCS8*/*9*/*14*/*15*

**DOI:** 10.3390/plants15193076

**Published:** 2026-10-08

**Authors:** Bo Shi, Yuanyuan Xie, Xuetong Yang, Feng Guan, Xinjian Wan

**Affiliations:** Jiangxi Key Laboratory of Horticultural Crops (Fruit, Vegetable & Tea) Breeding, Jiangxi Engineering Research Center of Vegetable Molecular Breeding, Jiangxi Academy of Agricultural Sciences, Institute of Vegetables and Flowers, Nanchang 330200, China; shibo_jiangxi@163.com (B.S.);

**Keywords:** *Benincasa hispida*, wax gourd, KCS gene family, cuticular wax, abiotic stress tolerance

## Abstract

The β-ketoacyl-CoA synthase (KCS) family catalyzes the rate-limiting step in very-long-chain fatty acid (VLCFA) synthesis and plays critical roles in cuticular wax formation and abiotic stress responses. However, the *KCS* gene family in wax gourd (*Benincasa hispida*) has remained uncharacterized. In this study, 27 *BhKCS* genes were identified genome-wide and unevenly distributed across ten chromosomes, with a dense cluster on chromosome 1. Phylogenetic analysis classified the BhKCS proteins into five groups together with *Arabidopsis* KCSs. Conserved motif and exon-intron structure analyses revealed that most members in Cluster I contain no introns or only one intron, whereas *BhKCS24–27* genes possess 14 exons. Promoter *cis*-element analysis showed that stress-responsive elements were the most abundant, followed by hormone-responsive and light-responsive elements, whereas development-related elements were relatively few. Subcellular localization showed that all four *BhKCS* fusion proteins localized to the cell membrane and co-localized with the plasma-membrane marker. Heterologous expression in yeast demonstrated that overexpression of BhKCS8 and BhKCS9 maintained yeast growth under osmotic, salt and acid stress conditions, whereas overexpression of BhKCS14 and BhKCS15 suppressed yeast growth under the same conditions, suggesting a functional dichotomy within the family in the yeast system. Scanning electron microscopy revealed that the powder wax gourd epidermis had a more continuous wax coating, whereas the green wax gourd epidermis showed a less continuous coating with larger areas of exposed epidermal surface. Expression profiling under cold, salt and drought stresses showed that several *BhKCS* genes exhibited variety-specific expression patterns between the green and powder wax gourd types, implying potential differences in their stress responsiveness. This study provides the first systematic characterization of the *BhKCS* gene family in wax gourd and identifies candidate genes for further functional validation.

## 1. Background

Plant cuticular wax serves as the first physical barrier between aerial plant organs and the environment, limiting non-stomatal water loss and protecting against UV radiation, pathogens, and abiotic stresses [1,2]. The wax layer, composed mainly of very-long-chain fatty acids (VLCFAs) and their derivatives, is essential for plant survival under adverse conditions such as drought, salinity, and extreme temperatures [3,4]. VLCFAs (≥C18) are direct precursors of cuticular wax and also play crucial roles in membrane lipids and suberin formation [5]. Their biosynthesis is catalyzed by an endoplasmic reticulum-localized fatty acid elongase complex, which adds two-carbon units sequentially to the growing acyl chain [6,7].

β-Ketoacyl-CoA synthase (KCS) is the rate-limiting and substrate-specific condensing enzyme of the elongase complex [8]. KCS proteins contain two conserved domains (FATTY ACID ELONGATION 1(FAE1)/type III PKS-like and Acyl carrier protein (ACP) synthase III C-terminal) and determine the final chain length of VLCFAs, thereby influencing plant stress tolerance [9]. The KCS gene family has been systematically characterized in many plants, including *Arabidopsis* (21 genes) [10], tomato (15) [11], pecan (30) [9], apple (28) [12], soybean (31) [13], and citrus [14]. Functional studies have shown that KCS genes are involved in cuticular wax synthesis, pollen development, and stress responses [15,16,17,18,19].

In *Arabidopsis thaliana*, *AtKCS1* is required for wax biosynthesis; its mutant exhibits reduced C26–C30 alcohols and aldehydes and increased drought sensitivity [20]. *AtKCS2* and *AtKCS20* are functionally redundant in cuticular wax and suberin production but are differentially regulated by osmotic stress [15]. *AtKCS6* (also named ECERIFERUM 6, CER6) is essential for the synthesis of C28–C32 fatty acids and is a major determinant of stem wax load [21]. *AtKCS10* (FIDDLEHEAD, FDH) affects floral organ development, while *AtKCS18* (FAE1) is crucial for seed VLCFA elongation [22,23]. These studies established that KCS genes control diverse aspects of plant growth, development, and stress adaptation through VLCFA chain-length regulation.

Multiple studies directly link KCS activity to abiotic stress tolerance. The *kcs1* mutant in *Arabidopsis* loses C26–C30 wax components and becomes more susceptible to drought [20]. Overexpression of the alfalfa KCS10 homologue *MsKCS10* increased cuticular wax crystal deposition in alfalfa leaves and in tobacco and tomato fruits and enhanced drought tolerance in transgenic tobacco [24]. In tomato, abscisic acid treatment induces *SlKCS8* and *SlKCS10* expression, and silencing these genes reduces proline accumulation and increases malondialdehyde content, indicating impaired osmotic adjustment and membrane integrity [11]. In pecan, CiKCS3/5/6/12-overexpressing yeast cells grew better under 5% polyethylene glycol (PEG), 300 mM NaCl and pH 4.0 conditions [9]. These results indicate that KCS-mediated VLCFA synthesis contributes directly to physical barrier reinforcement and also to signaling pathways that confer stress tolerance.

Wax gourd (*Benincasa hispida* Thunb.) is an economically important vegetable crop of the Cucurbitaceae family, widely cultivated across tropical and subtropical regions of Asia, particularly in China, India, and Southeast Asian countries [25]. However, wax gourd production is threatened by abiotic stresses such as drought, salinity, and low temperature, which negatively affect fruit quality and yield. The waxy coating on the fruit surface, which serves as a physical barrier against water loss, UV radiation and pathogen infection, has been shown to play a critical role in stress tolerance. Therefore, a better understanding of the genetic basis of wax formation in wax gourd has both fundamental and applied significance.

Here, we performed a genome-wide identification of the *BhKCS* gene family in wax gourd, analyzed their structural features and expression patterns under cold, salt and drought stresses, and compared the stress responses between green and powder wax gourd varieties. Our results provide the first systematic characterization of *BhKCS* genes in wax gourd and reveal potential candidates for improving stress tolerance and fruit quality in this economically important vegetable crop.

## 2. Results

### 2.1. Twenty-Seven BhKCS Genes Were Identified in the Wax Gourd

**Figure 1:**
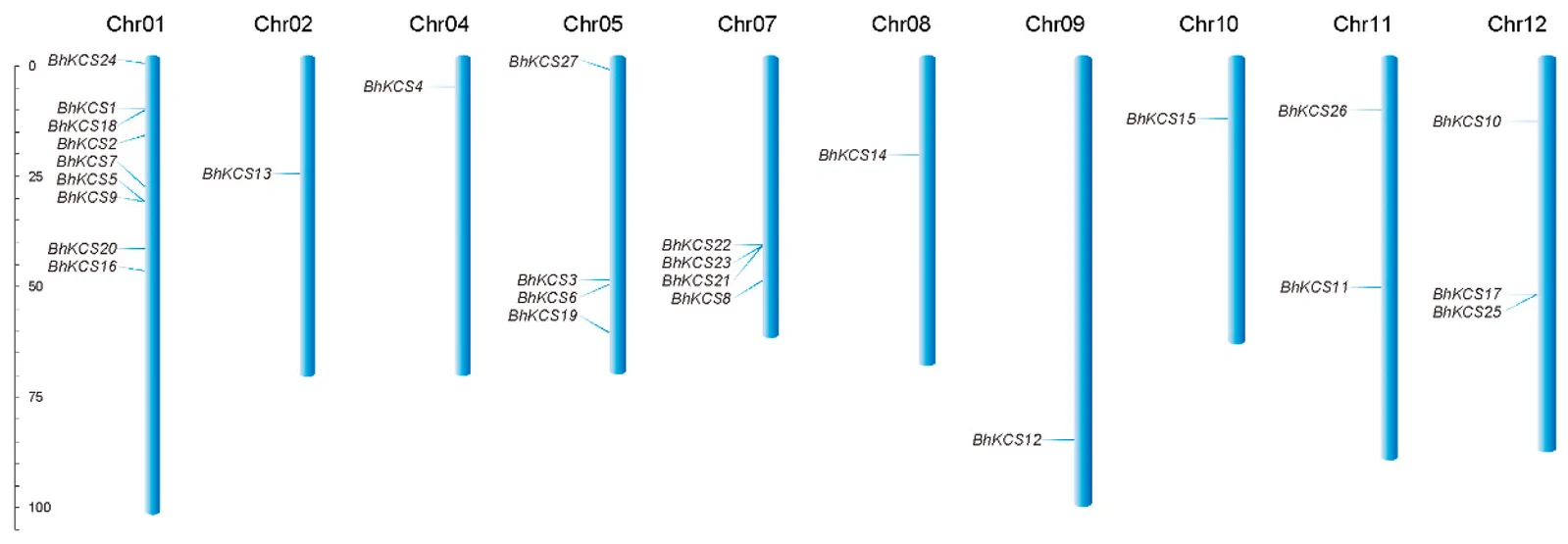
The gene locations of *BhKCS* gene family. The chromosome name is at the top of each bar. The scale of the chromosome is in millions of bases (Mb).

Twenty-seven BhKCS genes were identified in the wax gourd genome using HMMER3 and BLASTP tools, and they were designated BhKCS1 to BhKCS27 based on their chromosomal positions (Table 1). The proteins encoded by the BhKCS genes varied in length, with BhKCS23 being the shortest at 191 amino acids and BhKCS1 the longest at 528 amino acids. Their molecular weights ranged from 21,851.5 Da (BhKCS23) to 58,739.4 Da (BhKCS1), and the isoelectric points (pI) spanned from 5.94 to 9.85. In silico prediction indicated that BhKCS10 is predicted to localize to the chloroplast; BhKCS4 and BhKCS12 were predicted in both the chloroplast and cytoplasm; BhKCS23 in the cytoplasm and nucleus; BhKCS24, BhKCS26, and BhKCS27 in the peroxisome; and all other family members in the cytoplasm (Table 1). These values are computational predictions and are not necessarily identical to the experimentally determined localization (Section 2.5). These 27 genes were distributed across 10 chromosomes, with no members located on chromosomes 3 or 6. Among them, chromosome 1 harbored the highest number (9 members), while chromosomes 2, 4, 8, 9, and 10 each contained only one member (Figure 1). These findings reveal substantial variation in amino acid sequence length, isoelectric point, and molecular weight among the proteins encoded by distinct BhKCS family members.

### 2.2. Phylogenetic Classification of BhKCS Proteins

**Figure 2:**
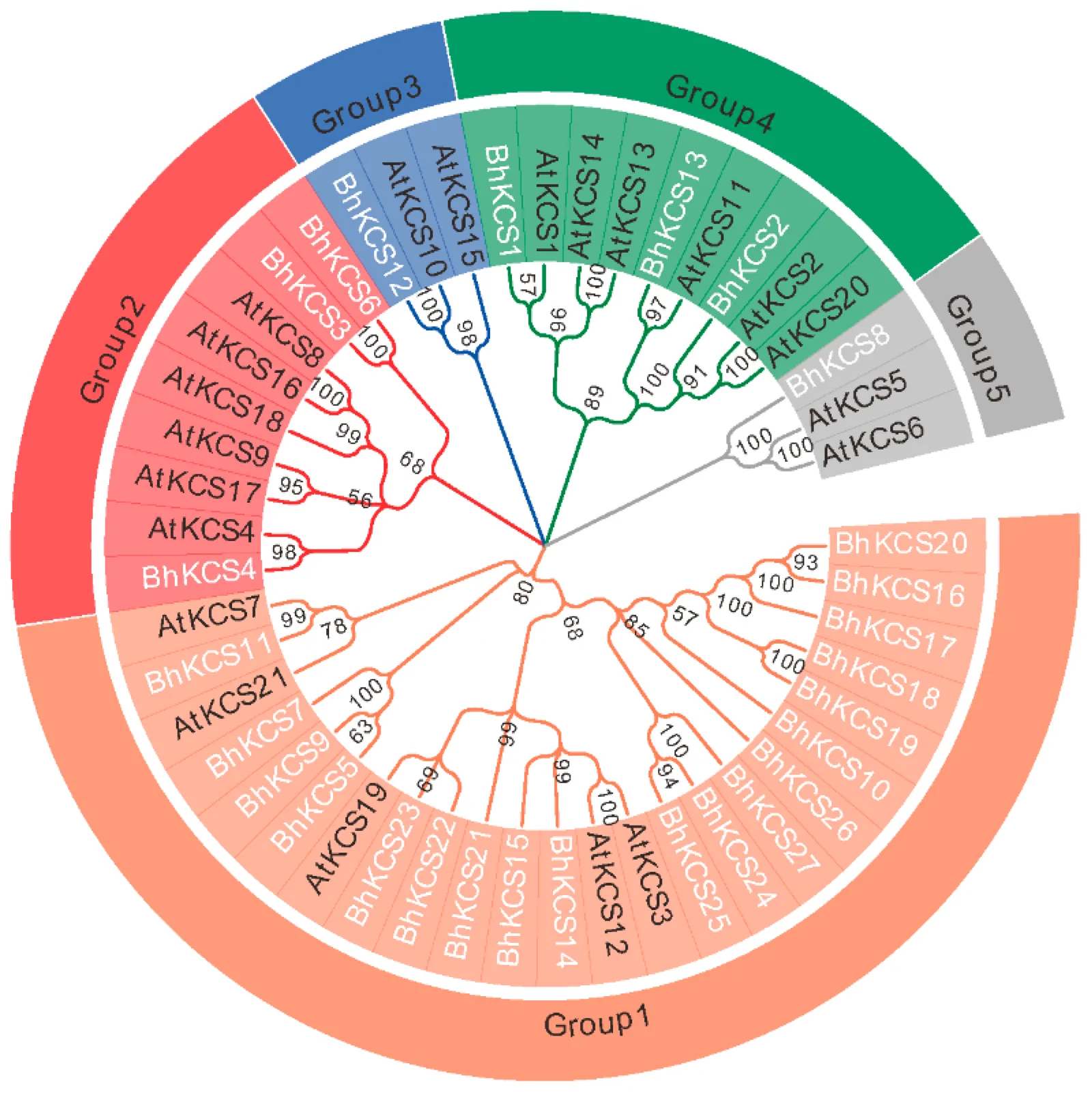
Molecular Phylogenetic analysis of KCS proteins from *B*. *hispida* and *A*. *thaliana*. The evolutionary history was inferred by using the Maximum Likelihood method based on the Whelan and Goldman model. The bootstrap consensus tree inferred from 1000 replicates is taken to represent the evolutionary history of the taxa analyzed. Branches corresponding to partitions reproduced in less than 50% bootstrap replicates are collapsed. Initial tree(s) for the heuristic search were obtained automatically by applying Neighbor-Join and BioNJ algorithms to a matrix of pairwise distances estimated using a JTT model, and then selecting the topology with superior log likelihood value. Evolutionary analyses were conducted in MEGA7.

To investigate the phylogenetic relationships of BhKCS proteins, a maximum-likelihood tree was constructed using KCS protein sequences from wax gourd (27 BhKCS) and *Arabidopsis thaliana* (21 AtKCS). As shown in Figure 2, all 48 KCS proteins were clearly divided into five distinct groups (Group1–Group5). Group5 contained three proteins: one *BhKCS* member (*BhKCS8*) and two AtKCS members. Group4 comprised 13 proteins: three *BhKCS* members (*BhKCS1*, *BhKCS2* and *BhKCS13*) and ten AtKCS members. Group3 consisted of three proteins: one *BhKCS* member (*BhKCS12*) and two AtKCS members. Group2 contained nine proteins: three *BhKCS* members (*BhKCS3*, *BhKCS4* and *BhKCS6*) and six AtKCS members. The remaining 19 *BhKCS* proteins, together with one AtKCS protein, formed Group1 (19 *BhKCS* plus 1 AtKCS member; 20 proteins in total), which represented the largest group (Figure 2). The distribution of BhKCS proteins across all five groups indicates potential functional divergence among members of the BhKCS gene family.

### 2.3. Conserved Motifs and Gene Structure of BhKCS Genes

**Figure 3:**
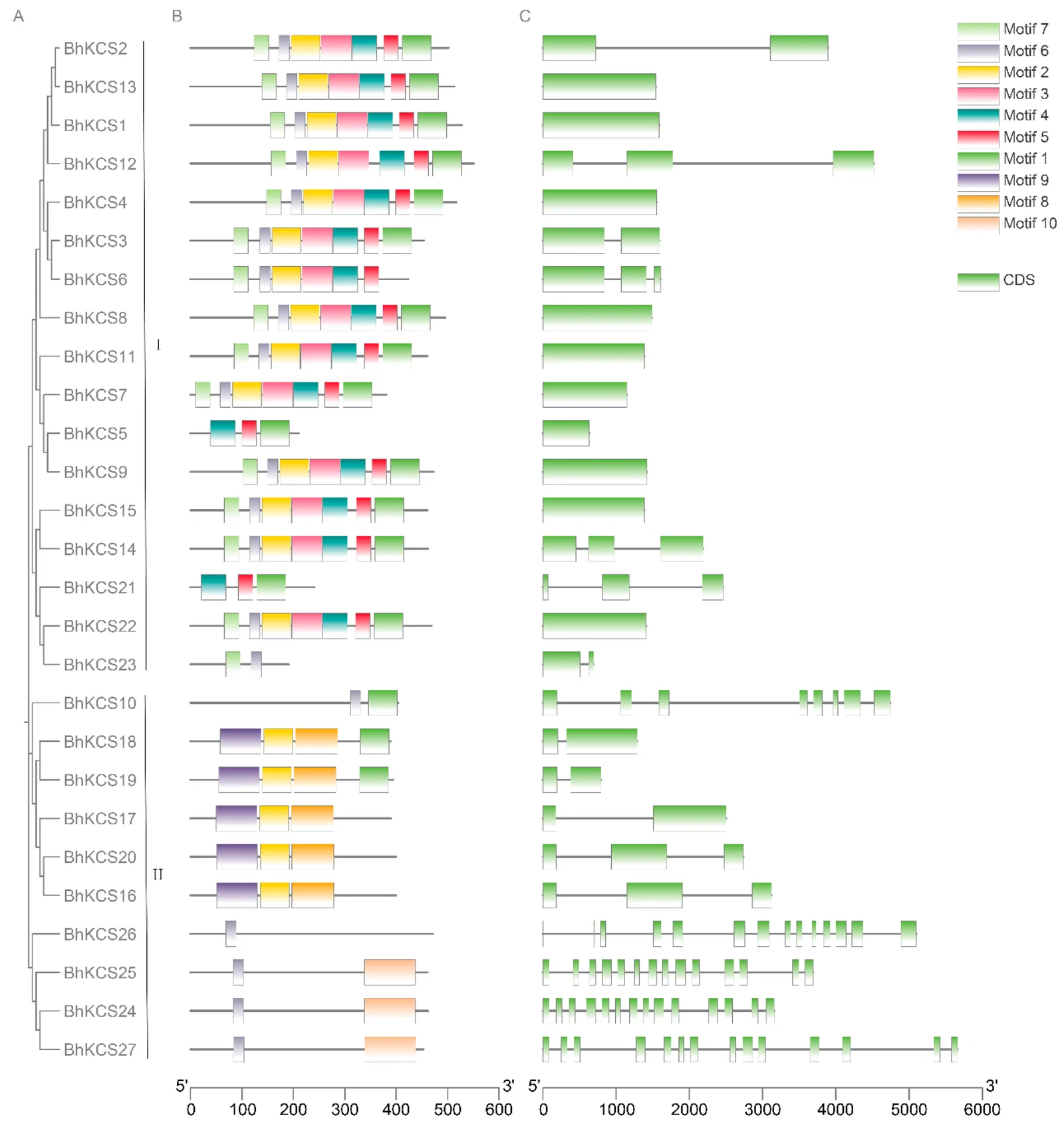
Gene structure of BhKCS. (**A**) Phylogenetic tree of BhKCS-encoding proteins. (**B**) The conserved motif distribution in the BhKCS protein sequence. (**C**) Genomic structure diagram of BhKCS in wax gourd. Exons are represented with green boxes; introns are shown with lines.

To further investigate the structural characteristics of the BhKCS gene family, conserved motif and gene structure analyses were performed. Based on the phylogenetic tree, the BhKCS genes were mainly divided into two clusters (Cluster I and Cluster II) (Figure 3A); this two-cluster division is derived from the BhKCS-only phylogeny and is distinct from the Group1–Group5 nomenclature used for the combined wax gourd–*Arabidopsis* tree shown in Figure 2. In Cluster I, most members contained seven conserved motifs (Motif1–Motif7), with the exception of BhKCS5 and BhKCS21, which possessed only three motifs (Motif1, Motif4, and Motif5), and BhKCS23, which contained only two motifs (Motif6 and Motif7) (Figure 3B). In Cluster II, BhKCS16–BhKCS20 predominantly harbored Motif2, Motif8, and Motif9, whereas BhKCS24–BhKCS27 mainly contained Motif6 and/or Motif10 (Figure 3B). Gene structure analysis revealed that the number of exons in Cluster I genes ranged from one to three. In Cluster II, BhKCS16–BhKCS20 had two or three exons, while BhKCS10 contained eight exons, and BhKCS24–BhKCS27 each possessed 14 exons (Figure 3C).

### 2.4. The Promoter Regions of BhKCS Genes Contain Various Cis-Elements

**Figure 4:**
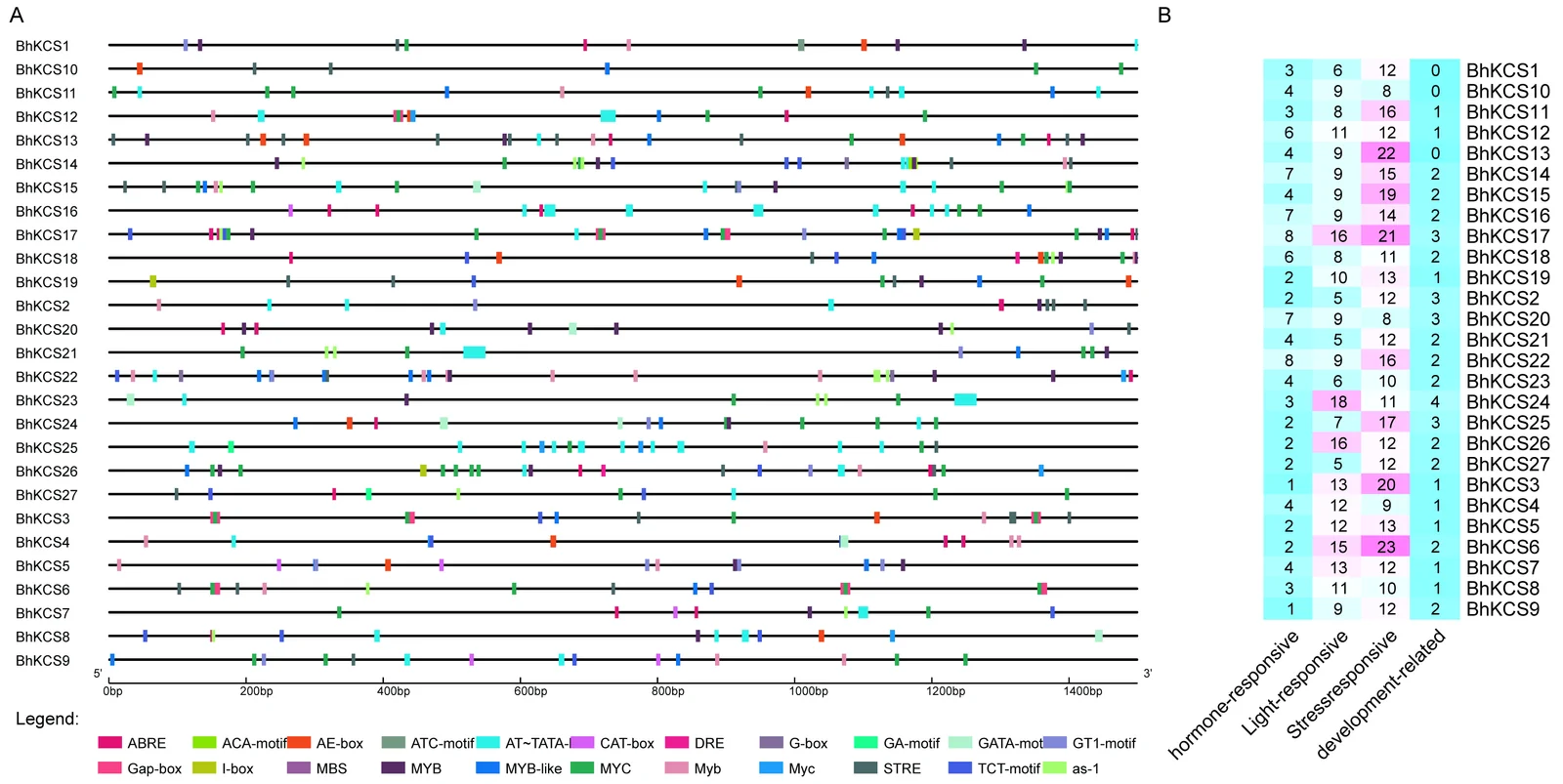
Prediction and analysis of cis-acting elements in the promoter regions (−1500 bp) of the *BhKCS* gene family. (**A**) *cis*-element distribution of *BhKCS* genes promoter. (**B**) Classification and statistics of cis-elements. The elements are categorized into four major types. hormone-responsive: ABRE, ABRE3a, ABRE4, AuxRR-core, CGTCA-motif, GARE-motif, P-box, TATC-box, TCA-element, TGA-element, TGACG-motif; Light-responsive: 3-AF1 binding site, ACE, AE-box, AT1-motif, ATCT-motif, Box 4, Box II, Box III, chs-CMA1a, chs-CMA2a, G-box, Gap-box, GATA-motif, GT1-motif, I-box, LAMP-element, MRE, TCT-motif, TCCC-motif; Stress-responsive: ARE, as-1, DRE core, ERE, LTR, MBS, MYB, MYB-like sequence, MYB recognition site, MYC, STRE, TC-rich repeats, W box, WRE3, WUN-motif; development-related: AACA_motif, AT-rich element, AT-rich sequence, CAT-box, circadian, F-box, GCN4_motif, MSA-like, O2-site.

Analysis of the 1500 bp upstream region of the BhKCS gene promoters revealed distinct cis-acting elements (Figure 4A). These elements were classified into four functional categories: hormone-responsive, light-responsive, stress-responsive, and plant growth and development-related elements. Among the 27 members, all except BhKCS1, BhKCS10, and BhKCS13 contained all four types of cis-acting elements. Notably, light-responsive and stress-responsive elements were particularly abundant, suggesting that *BhKCS* family members may be highly involved in stress responses (Figure 4B).

### 2.5. Subcellular Localization of BhKCS8/9/14/15

**Figure 5:**
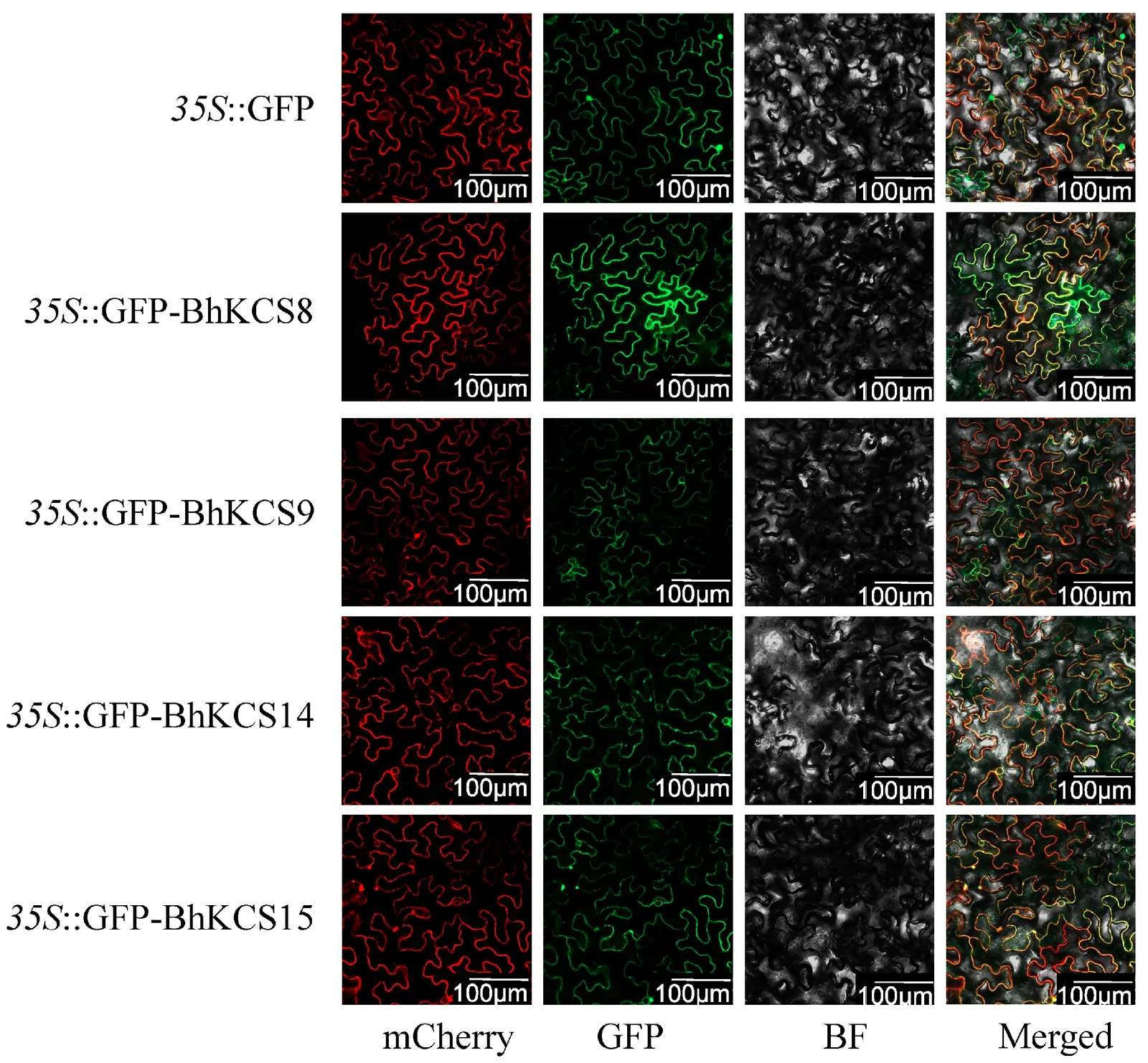
Subcellular localization of four BhKCS proteins. The transient expression of the 35S::BhKCS-GFP fusion construct and the 35S::GFP construct in *N*. *benthamiana*. Green fluorescent protein (GFP) fluorescence was observed using a confocal microscope. To visualize the plasma membrane, a plasma-membrane marker consisting of Aquaporin PIP2-1 fused to mCherry (35S::PIP2-1-mCherry) was co-expressed for counterstaining. Each construct was infiltrated separately into independent leaf areas, and each image is representative of at least three independent infiltration events; similar plasma-membrane localization patterns were observed in all imaged cells. Scale bars = 100 µm.

Subcellular localization prediction provides insights into the functional distribution of proteins within plant cells, facilitating a better understanding of their roles at the protein level. According to the prediction results, BhKCS8, BhKCS9, BhKCS14, and BhKCS15 were localized in the cytoplasm. Notably, BhKCS8 and BhKCS9 showed high similarity to *Arabidopsis* AtKCS6, while BhKCS14 and BhKCS15 were closely related to AtKCS3 and AtKCS12, suggesting their potential involvement in cuticular wax synthesis. Moreover, the promoter regions of these four genes contained multiple stress-responsive cis-acting elements. Based on these findings, BhKCS8, BhKCS9, BhKCS14, and BhKCS15 were selected for subcellular localization analysis. The results showed that the 35S::GFP control protein was present in the plasma membrane, cytoplasm, and nucleus. The fluorescence signals of all four fusion proteins (35S::BhKCS8-GFP, 35S::BhKCS9-GFP, 35S::BhKCS14-GFP and 35S::BhKCS15-GFP) were predominantly detected at the cell periphery and co-localized with the plasma-membrane marker. In contrast to the free GFP control, no nuclear signal was observed for any of the four BhKCS-GFP fusion proteins, indicating that BhKCS8, BhKCS9, BhKCS14 and BhKCS15 are localized to the cell membrane (Figure 5).

### 2.6. Effects of BhKCS8/9/14/15 Overexpression on Yeast Growth Under Osmotic, Salt and Acid Stress Treatments

**Figure 6:**
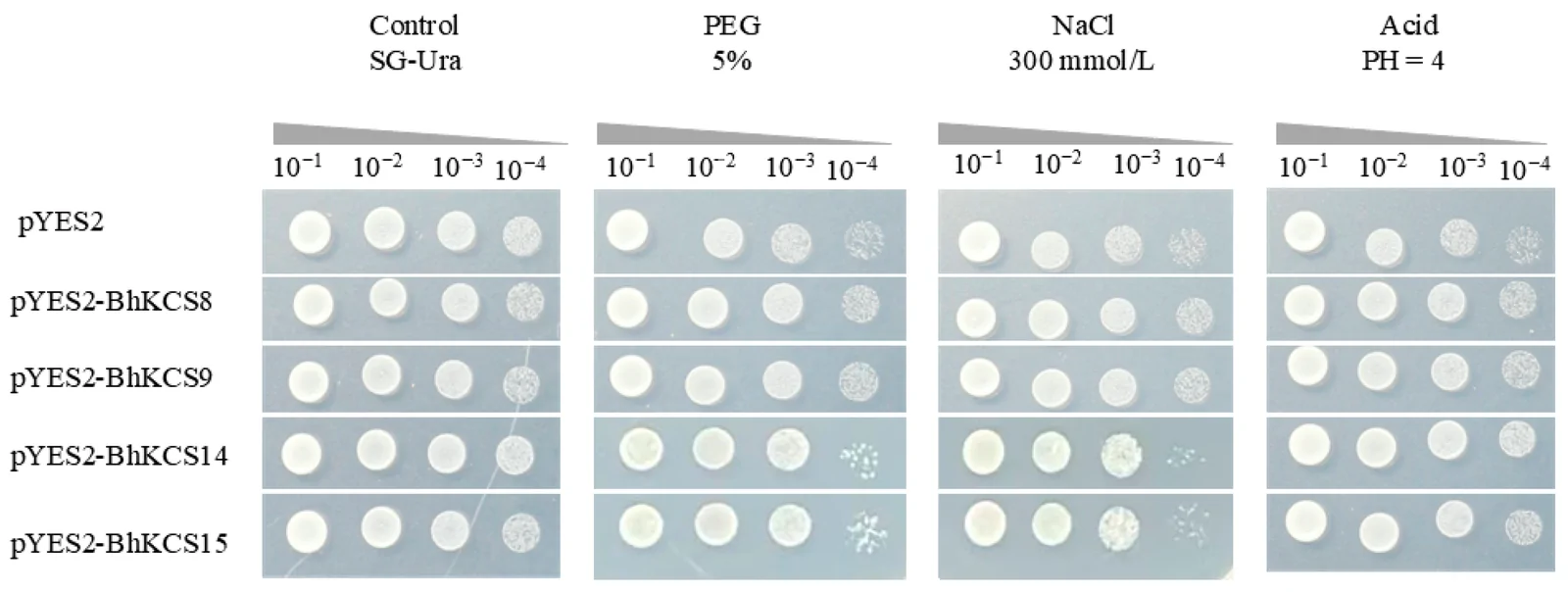
Stress tolerance analysis of BhKCS8, BhKCS9, BhKCS14 and BhKCS15-overexpressing yeast cells. Yeast cells containing pYES2-BhKCS8, pYES2-BhKCS9, pYES2-BhKCS14, pYES2-BhKCS15 and an empty pYES2 vector (control) were incubated in SG/-Ura liquid medium containing 2% (*w*/*v*) galactose for 3–5 d. Serial ten-fold dilutions (10^−1^–10^−4^) of each culture were then spotted onto SG/-Ura plates containing 5% (*w*/*v*) PEG6000 (≈−0.05 MPa, −0.5 bar), 300 mM NaCl (≈−1.5 MPa, −15 bar), or adjusted to pH 4.0 with HCl. A representative plate from three independent experiments is shown.

BhKCS8, BhKCS9, BhKCS14, and BhKCS15 were predicted to play important roles in cuticular wax synthesis, and their promoter regions contained multiple stress-responsive cis-acting elements. To investigate whether these genes are involved in abiotic stress responses, the four recombinant constructs along with the empty vector (pYES2) as a control were transformed into yeast cells. After obtaining the overexpressing yeast lines, three types of abiotic stress treatments were applied. Under stress conditions, the pYES2 empty vector control group showed reduced growth at the 10^−3^ and 10^−4^ dilution concentrations compared to the untreated control. In contrast, yeast cells overexpressing *BhKCS8* and *BhKCS9* exhibited no significant changes in growth status under stress at the same dilutions, indicating that their overexpression did not impair cellular tolerance to these stresses. However, yeast cells overexpressing *BhKCS14* and *BhKCS15* displayed reduced growth under salt and osmotic stress conditions (Figure 6). To distinguish the responses to osmotic and salt stress of different intensities, the osmotic potential of each treatment was calculated using the empirical equation of Michel and Kaufmann [26]. At 25 °C, the 5% (*w*/*v*) PEG6000 treatment corresponded to approximately −0.05 MPa (−0.5 bar), representing a mild osmotic challenge, whereas the 300 mM NaCl treatment corresponded to approximately −1.5 MPa (−15 bar), representing a substantially stronger salt stress; the acid treatment (pH 4.0) provided an additional, non-osmotic type of stress. These observations describe the growth phenotypes of the heterologously expressed BhKCS proteins in yeast and are indicative of differential responses in this heterologous system rather than of their physiological function in planta.

### 2.7. Microscopic Observation of Cuticular Wax Morphology in Green Wax Gourd and Powder Wax Gourd

**Figure 7:**
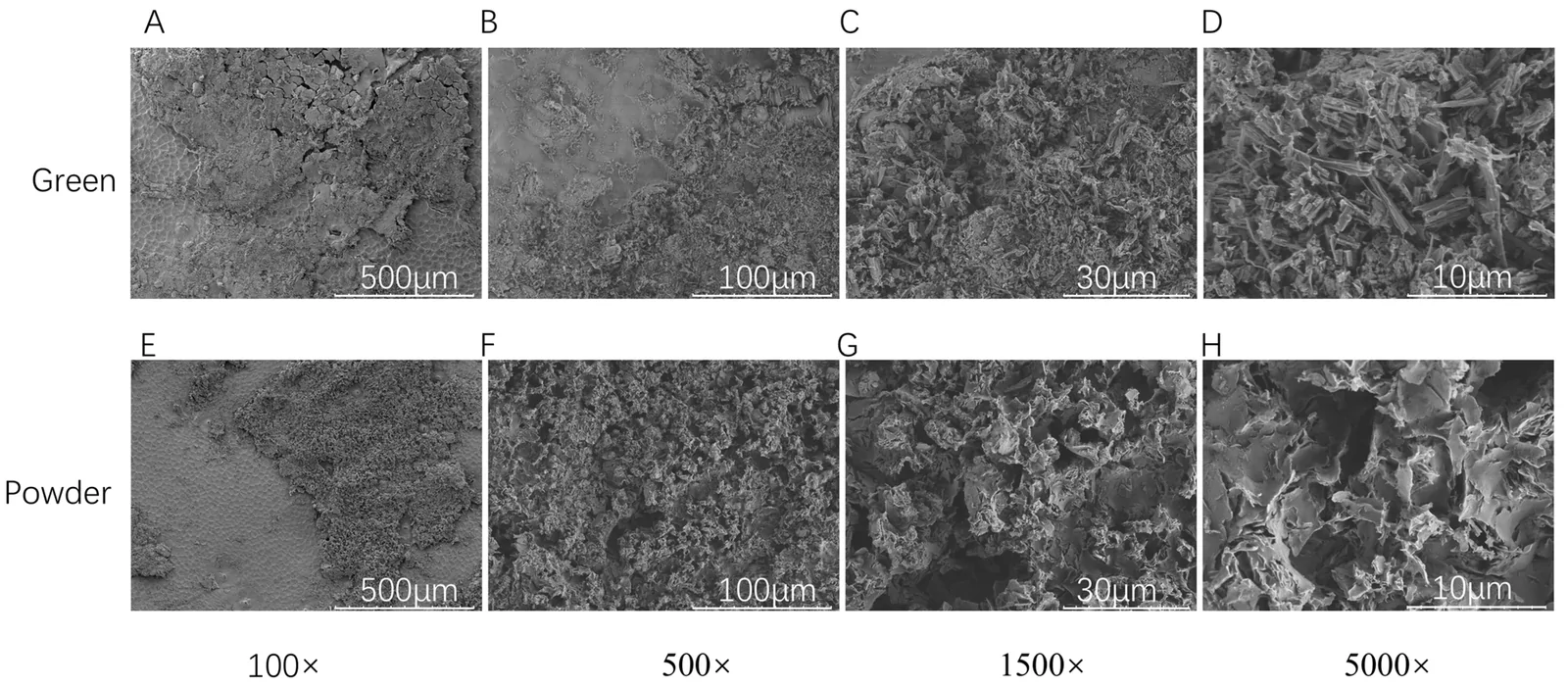
Scanning electron micrographs of the exocarp surface of green wax gourd and powder wax gourd. (**A**–**D**) Green wax gourd; (**E**–**H**) powder wax gourd. (**A**,**E**) 100×, scale bar = 500 µm; (**B**,**F**) 500×, scale bar = 100 µm; (**C**,**G**) 1500×, scale bar = 30 µm; (**D**,**H**) 5000×, scale bar = 10 µm. Panels (**A**–**D**) and (**E**–**H**) are successive magnifications of the same field of view of the exocarp surface; the micrographs shown are representative of three independent biological samples per type, and no quantitative statistics were applied to these qualitative images.

Two common cultivated types of wax gourd, green wax gourd and powder wax gourd, exhibit marked differences in the microstructure of their surface waxes. Scanning electron microscopy (SEM) revealed that the two types differ in the extent and continuity of their surface wax coverage. The powder wax gourd epidermis showed a more continuous, densely granular wax coating, whereas the green wax gourd epidermis showed a less continuous coating with larger areas of exposed epidermal surface (Figure 7). Rod-like wax structures were observed on both types at higher magnification, but their abundance varied among fields of view.

### 2.8. Expression Profiles of BhKCS Genes Under Cold, Salt, and Drought Stresses in Green Wax Gourd and Powder Wax Gourd

**Figure 8:**
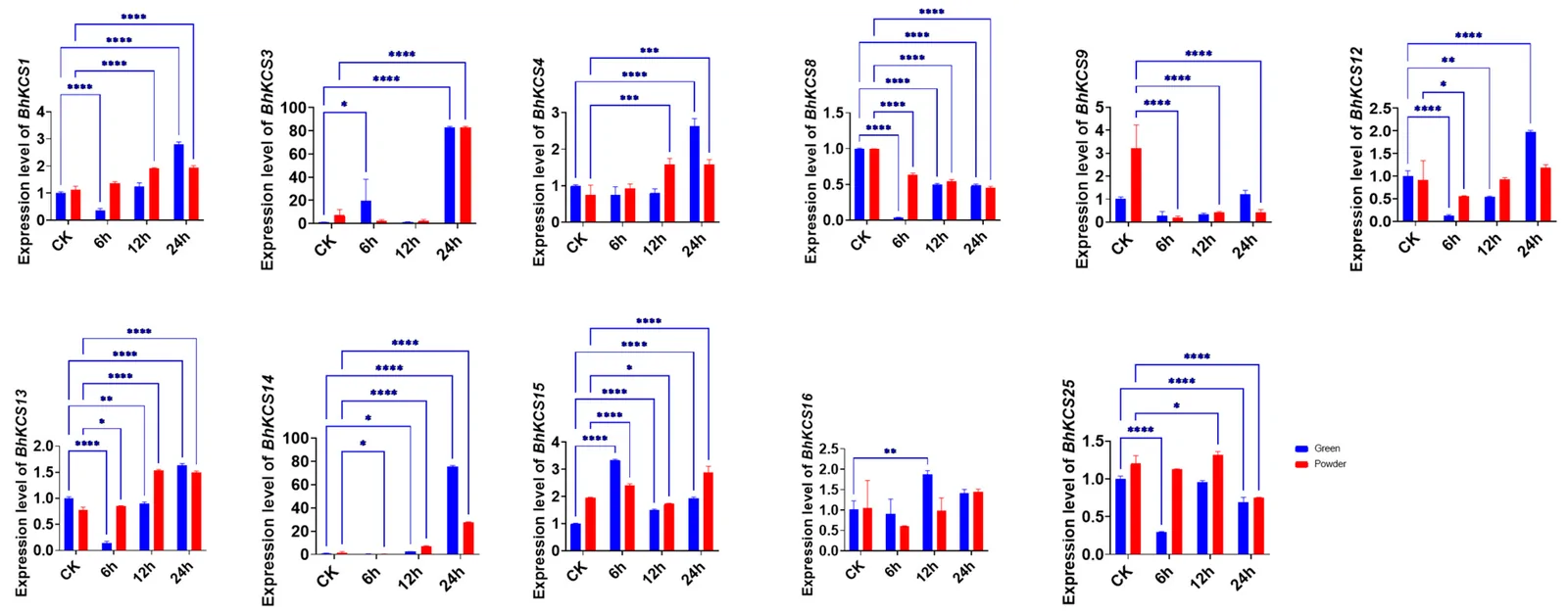
Expression profiles of eleven *BhKCS* genes (*BhKCS1*, *BhKCS3*, *BhKCS4*, *BhKCS8*, *BhKCS9*, *BhKCS12*, *BhKCS13*, *BhKCS14*, *BhKCS15*, *BhKCS16* and *BhKCS25*) in green wax gourd and powder wax gourd under cold stress. Samples were taken after treatments for 0 h, 6 h, 12 h, and 24 h. All data are presented as the mean (±SD) of three independent biological determinations and were analyzed by two-way ANOVA (* *p* < 0.05, ** *p* < 0.01, *** *p* < 0.001, **** *p* < 0.0001).

**Figure 9:**
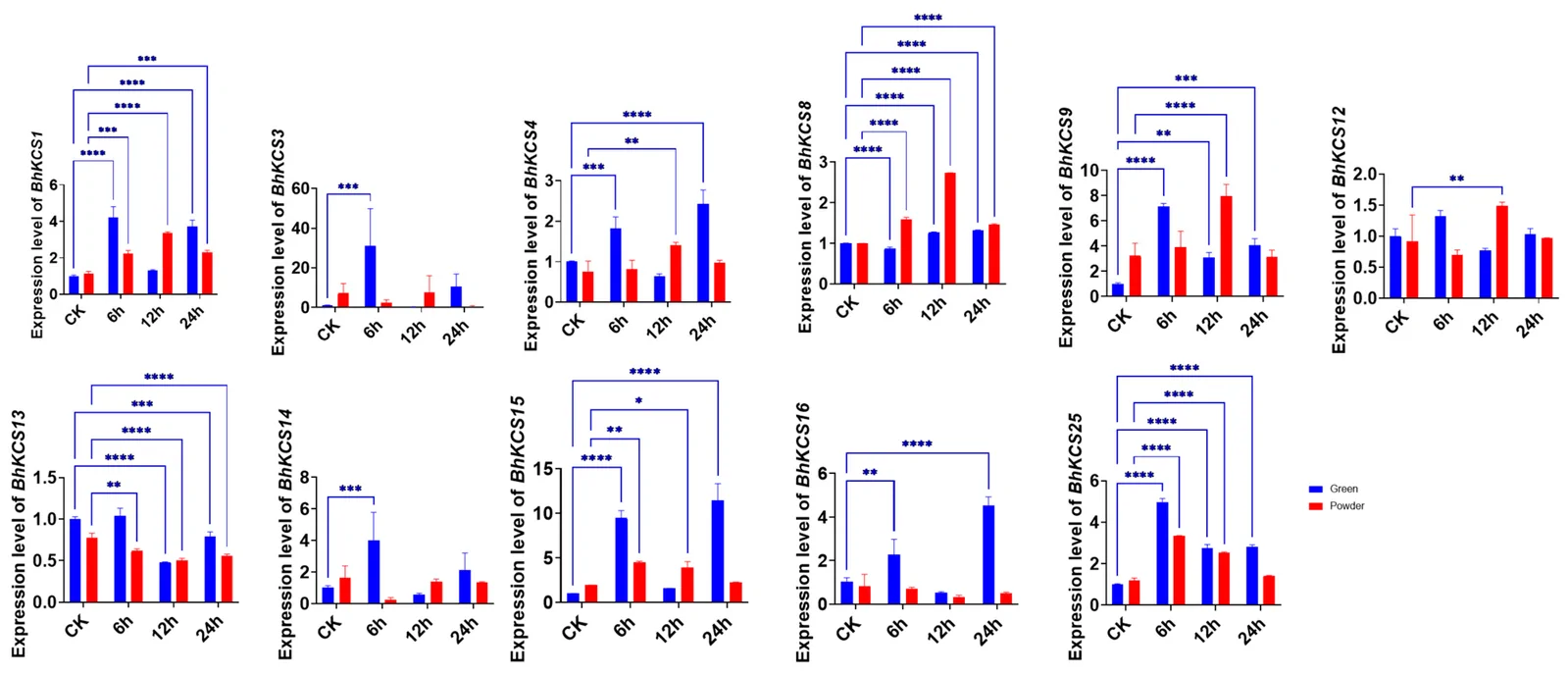
Expression profiles of eleven *BhKCS* genes (*BhKCS1*, *BhKCS3*, *BhKCS4*, *BhKCS8*, *BhKCS9*, *BhKCS12*, *BhKCS13*, *BhKCS14*, *BhKCS15*, *BhKCS16* and *BhKCS25*) in green wax gourd and powder wax gourd under salt stress treatment. Samples were taken after treatments for 0 h, 6 h, 12 h, and 24 h. All data are presented as the mean (±SD) of three independent biological determinations and were analyzed by two-way ANOVA (* *p* < 0.05, ** *p* < 0.01, *** *p* < 0.001, **** *p* < 0.0001).

**Figure 10:**
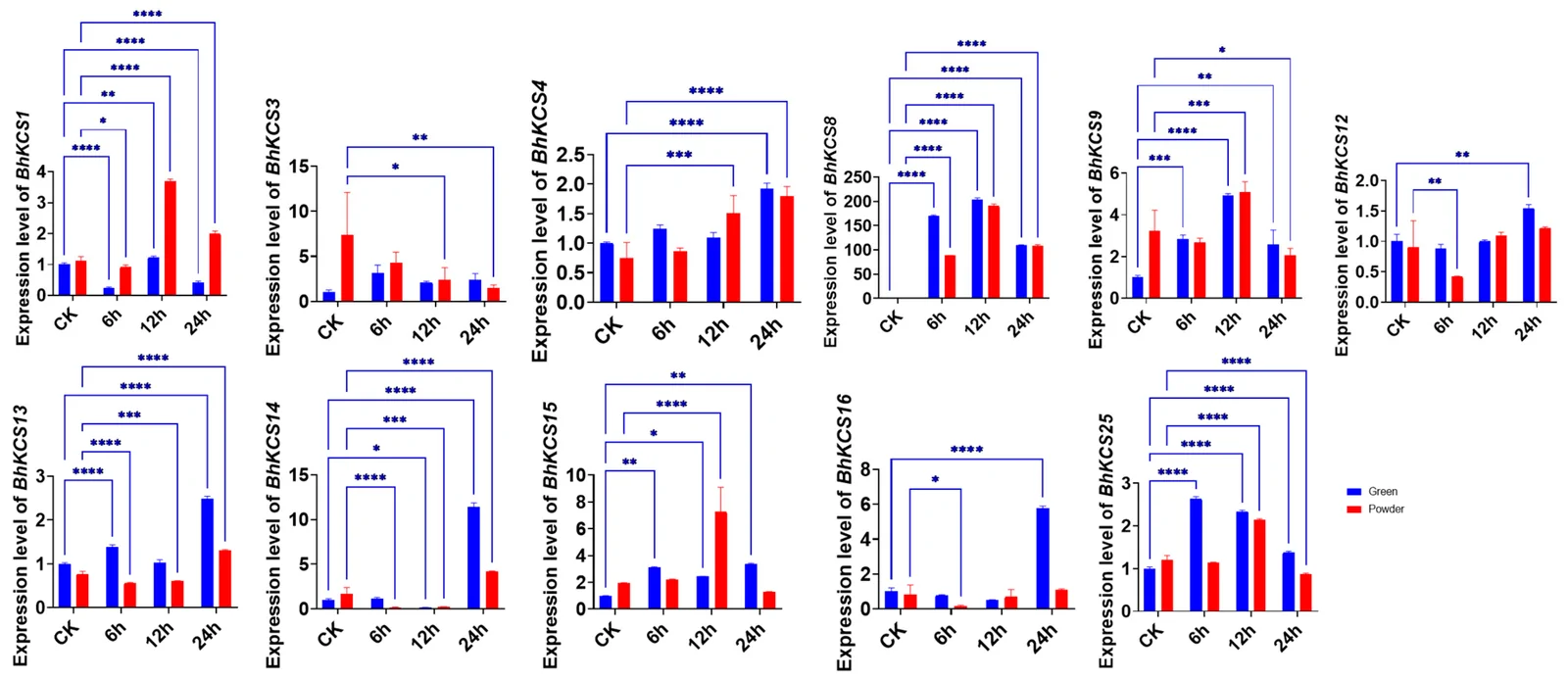
Expression profiles of eleven *BhKCS* genes (*BhKCS1*, *BhKCS3*, *BhKCS4*, *BhKCS8*, *BhKCS9*, *BhKCS12*, *BhKCS13*, *BhKCS14*, *BhKCS15*, *BhKCS16* and *BhKCS25*) in green wax gourd and powder wax gourd under drought stress treatment. Samples were taken after treatments for 0 h, 6 h, 12 h, and 24 h. All data are presented as the mean (±SD) of three independent biological determinations and were analyzed by two-way ANOVA (* *p* < 0.05, ** *p* < 0.01, *** *p* < 0.001, **** *p* < 0.0001).

To investigate whether the *BhKCS* genes respond differently to abiotic stress between the two wax gourd types that differ in surface wax morphology, plants were subjected to cold, salt, and drought stress treatments, and the expression levels of eleven *BhKCS* genes (*BhKCS1*, *BhKCS3*, *BhKCS4*, *BhKCS8*, *BhKCS9*, *BhKCS12*, *BhKCS13*, *BhKCS14*, *BhKCS15*, *BhKCS16*, and *BhKCS25*) were examined. These genes were selected based on their phylogenetic relationships, promoter cis-element composition, and potential involvement in stress responses. Under cold stress, the expression of *BhKCS1*, *BhKCS3*, *BhKCS4*, *BhKCS13*, and *BhKCS14* gradually increased between 6 h and 24 h after treatment, whereas *BhKCS8* and *BhKCS9* showed a gradual decrease. Notably, the expression patterns of certain genes differed markedly between the two varieties: the expression levels of *BhKCS8*, *BhKCS12*, *BhKCS13*, and *BhKCS25* decreased more sharply in green wax gourd at 6 h post-treatment, while *BhKCS15* showed a greater increase in green wax gourd at the same time point (Figure 8). Under salt stress, the expression of *BhKCS1*, *BhKCS4*, *BhKCS8*, *BhKCS9*, *BhKCS15*, and *BhKCS25* gradually increased from 6 h to 24 h, whereas *BhKCS13* showed a decreasing trend; *BhKCS3*, *BhKCS14*, and *BhKCS25* exhibited an initial increase followed by a decrease. Furthermore, the expression of several genes, including *BhKCS1*, *BhKCS3*, *BhKCS9*, *BhKCS14*, *BhKCS15*, and *BhKCS25*, was more dramatically affected in green wax gourd at 6 h after salt treatment (Figure 9). Under drought stress, *BhKCS1*, *BhKCS4*, *BhKCS8*, *BhKCS9*, *BhKCS14*, *BhKCS15*, *BhKCS16*, and *BhKCS25* showed upregulation at multiple time points between 6 h and 24 h; *BhKCS3* expression increased in green wax gourd but decreased in powder wax gourd. In addition, the upregulation of *BhKCS14* and *BhKCS16* at 24 h post-drought was greater in green wax gourd than in powder wax gourd, while the upregulation of *BhKCS15* at 12 h was stronger in powder wax gourd than in green wax gourd (Figure 10). Taken together, these expression patterns suggest that most of these BhKCS genes are involved in the response of wax gourd to abiotic stresses, and that the two varieties exhibit distinct transcriptional responses.

## 3. Discussion

Wax gourd (*Benincasa hispida*) exhibits a distinct cuticular wax bloom on the fruit surface, yet the underlying *KCS* gene family remains uncharacterized. Here, 27 *BhKCS* genes were identified, phylogenetically classified into five groups, and showed diverse structures. Yeast assays revealed that BhKCS8/9 did not impair growth under osmotic, salt and acid stress, whereas BhKCS14/15 suppressed growth, indicating a functional dichotomy within the family in the yeast system. Expression profiling under cold, salt and drought revealed variety-specific transcriptional responses under stress.

The KCS family members are primarily involved in the synthesis of very-long-chain fatty acids (VLCFAs). In this study, a phylogenetic analysis of KCS proteins from wax gourd (*Benincasa hispida*) and *Arabidopsis thaliana* was performed, and the results showed that the family could be divided into five groups, which is highly similar to the grouping of KCS members in sorghum [27]. Although different authors have defined subfamily assignments of KCS members in their studied species with some variations, the clustering of homologous members remains highly consistent across studies [9,11,12,13,14,27,28,29,30]. A total of 27 KCS members were identified in wax gourd. This number represents a slight increase compared with the 21 members in *Arabidopsis*, which may be attributed to the larger genome size of wax gourd [25]. Interestingly, the *KCS* genes were not evenly distributed across chromosomes; nine members were concentrated on chromosome 1. Several of these members tend to form clusters, suggesting high sequence similarity and possible segmental duplication events during their evolution. In contrast, the number of members on other chromosomes ranged from only 0 to 4 (Figure 1).

Genes with similar gene structures and protein structures usually share similar functions. Among the 27 family members in wax gourd, particularly those in Cluster I (Figure 3A), most of the open reading frames contain no introns or only one intron. This structural feature is very similar to the gene structure patterns observed in *Arabidopsis* and other species [10], further indicating that members within the same cluster possess comparable functions. Cuticular wax is deposited progressively onto the surface of developing organs as epidermal cells emerge and expand [31], and the distribution and amount of cuticular wax are closely associated with plant stress tolerance [1,2]. In this study, analysis of the cis-acting regulatory elements in the promoters of the 27 *BhKCS* genes revealed that, among the four types of regulatory elements, stress-responsive elements were the most abundant, whereas development-related elements were relatively fewer. This further supports the involvement of these genes in the regulation of stress responses.

In *Arabidopsis*, AtKCS1, AtKCS2, AtKCS5 and AtKCS6, which are distributed in groups 4 and 5, are mainly involved in cuticular wax and suberin synthesis [15,17,20,21], whereas AtKCS3, AtKCS12 and AtKCS19 form a separate cluster. Among these, AtKCS3 acts as a negative regulator of wax synthesis through protein–protein interaction with AtKCS6 [18]. In addition, AtKCS12 and AtKCS19 participate in seed VLCFA accumulation and exhibit tissue-specific expression patterns [19]. BhKCS8 clustered with AtKCS5/AtKCS6, suggesting a possible positive role in cuticular wax synthesis and stress responses. In contrast, BhKCS14 and BhKCS15, which clustered with AtKCS3 and AtKCS12, may have distinct or even opposing functions. This hypothesis is consistent with our yeast heterologous expression results, in which BhKCS8/9-overexpressing yeast cells showed no growth impairment under stress, whereas BhKCS14/15-overexpressing cells showed growth inhibition. Nevertheless, these yeast-based observations do not constitute definitive conclusions about their regulatory roles in wax gourd and require further genetic and physiological evidence.

In agricultural production, wax gourd is mainly divided into two types: green wax gourd and powder wax gourd. From a geographical distribution perspective, green wax gourd appears to be grown in warmer southern regions (e.g., Guangdong, Guangxi, Hainan provinces), whereas powder wax gourd is mainly distributed in more northern areas such as Yunnan and Sichuan provinces. This differential distribution may be related to temperature stress. In our cold stress experiment, the expression of some members, such as *BhKCS9*, was significantly reduced in powder wax gourd, while *BhKCS16* showed a significant increase only in green wax gourd after cold treatment (Figure 8). Under salt stress, the expression levels of *BhKCS3*, *BhKCS14* and *BhKCS16* showed an increasing trend exclusively in green wax gourd, indicating a marked varietal difference (Figure 9). Under drought stress, the expression of *BhKCS3* decreased significantly only in powder wax gourd (Figure 10). Whether these genes are functionally related to the amount of cuticular wax on the fruit surface and to stress tolerance still requires further experimental evidence.

## 4. Materials and Methods

### 4.1. Plant Materials and Growth Conditions

The experimental materials used were wax gourd variety ‘Zaoyoufen’ (Powder wax, abbreviated as “P”), developed at the Vegetables and Flowers Research Institute of the Jiangxi Academy of Agricultural Sciences and ‘Lp20210317’ (Green wax, abbreviated as “G”), provided by the Jiangxi Academy of Agricultural Sciences, China. After soaking in water for 24 h, the seeds were germinated at 32 °C for 48 h at 80% relative humidity to prevent dehydration. The germinated seeds were sown in seedling trays filled with nutrient soil. The core component of nutrient soil was commercially available Pindstrup seedling-specific peat (Denmark) (Model: 505+, particle size: 0–6 mm, pH 6.0) was sourced from Pindstrup Mosebrug A/S, Ry, Denmark. Subsequently, it was mixed and formulated with other modified substrates at the following volume ratio: 60% Pindstrup peat + 25% perlite + 15% vermiculite. One seed was sown per cell of the tray and then covered with a 1–2 cm thick layer of nutrient soil. Subsequently, the seedling trays were placed in a growth chamber for cultivation, with the cultivation conditions of 30 °C/28 °C (day/night), a 14 h/10 h photoperiod, and a light intensity of 10,000 lx provided by white LED light sources (correlated color temperature ≈ 6000 K), corresponding to a photosynthetic photon flux density (PPFD) of approximately 150 µmol m^−2^ s^−1^, at a relative humidity of 70%. The substrate was sterilized by conventional moist-heat (autoclave) sterilization, and its electrical conductivity was not measured.

### 4.2. Identification of BhKCS Genes in Wax Gourd

This study employed a comprehensive approach to identify all members of the KCS gene family in wax gourd (*Benincasa hispida*). The chromosome-level genome assembly and its annotation files for wax gourd were downloaded from the National Genomics Data Center (NGDC) (accession number: GWHBJVO00000000) or the National Center for Biotechnology Information (NCBI) (accession number: GCA_027475165.1) [25]. Candidate KCS genes were initially identified using a dual-strategy search. First, a local BLASTP (v2.0) analysis was performed using the complete set of *Arabidopsis thaliana* KCS protein sequences as queries against the predicted wax gourd proteome. Second, a Hidden Markov Model (HMM) search was conducted on the same proteome using HMMER 3.0 software with the KCS-specific HMM profiles PF08392 and PF08541 (e-value cutoff ≤ 1 × 10^−5^). The intersection of the protein sets obtained from these two independent searches formed the preliminary candidate list. To authenticate these candidates, the protein sequences were subsequently analyzed for the presence of definitive KCS conserved domains using the Pfam, SMART, and NCBI’s Conserved Domain Database (CDD). Only proteins that contained the full complement of characteristic domains across all three databases were retained as bona fide KCS family members for subsequent analysis.

### 4.3. Sequence and Phylogenetic Analysis

For an in-depth characterization of the *KCS* genes in wax gourd, comprehensive bioinformatic analyses were undertaken. The evolutionary relationships were elucidated by constructing a phylogenetic tree. Full-length KCS protein sequences from *Benincasa hispida* and *Arabidopsis thaliana* were aligned using ClustalW (v1.83), and the phylogenetic tree was generated in MEGA 7.0 under the Maximum Likelihood method with the Whelan and Goldman (WAG) model, supported by 1000 bootstrap replicates [32]. The genomic architecture of the *BhKCS* genes was analyzed for exon-intron organization using the TBtools software (v2.487) [33]. To identify conserved sequence patterns, protein motif analysis was performed with the MEME tool, configured to discover up to 10 motifs, and the significance of these motifs was visualized via Weblogo (https://weblogo.berkeley.edu/create.cgi, accessed on 26 September 2026). Regulatory potential was assessed by extracting 1.5 kb upstream sequences of each *BhKCS* gene as putative promoters and scanning them for *cis*-regulatory elements with the PlantCARE database [34]. The subcellular destinations of BhKCS proteins were computationally predicted using Plant-mPLoc [35]. Finally, the genomic positions of all *BhKCS* genes were determined from the chromosome-level assembly and visualized on chromosomes using MapInspect software (v1.0).

### 4.4. Wax Gourd Stress Treatment

Two wax gourd inbred lines, P and G, were used in this study. When seedlings reached the four-leaf and one-heart stage (average height 14.66 cm and an average of 6.5 leaves per plant), they were subjected to three types of abiotic stress: low temperature (4 °C), drought (simulated by 5% (*w*/*v*) PEG6000), and salt stress (150 mmol/L NaCl). Each treatment comprised 96 plants per genotype and was applied by drenching in 32-cell plug trays. For each stress treatment, leaf samples were collected at 0 h (control, before stress), 6 h, 12 h, and 24 h after treatment. Each time point included three independent biological replicates. All samples were immediately frozen in liquid nitrogen and stored at −80 °C for subsequent analysis.

### 4.5. RNA Extraction and Real-Time Quantitative PCR Analysis

Total RNA was extracted from different plant materials using RNA Plant Kit (Takara, Qingdao, China) and treated with DNase I (Takara, Qingdao, China) to remove genomic DNA. For RNA extraction, a Centrifuge 5424 R refrigerated microcentrifuge (Eppendorf, Hamburg, Germany) was used, and RNA quality and concentration were assessed with a NanoDrop One spectrophotometer (Thermo Fisher Scientific, Waltham, MA, USA). Reverse transcription was performed using the HiScript II 1st strand complementary DNA (cDNA) synthesis kit (Vazyme, Nanjing, China). qRT-PCR was performed on a LightCycler^®^ 480 Real-Time PCR System (Roche, Basel, Switzerland) using a 20 µL reaction volume comprising 10 µL SYBR qPCR Master Mix (Vazyme, Nanjing, China), 6.4 µL of ddH_2_O, 0.8 µL of forward primer (10 mmol/L), 0.8 µL of reverse primer, and 2 µL of template cDNA. *BhUBQ* was used as the internal reference gene for qRT-PCR [36]. The relative expression patterns of the *BhKCS* genes between the two wax gourd types and across the stress time course were used as the primary readout of this screening analysis. The gene-specific primers are listed in Supplementary Table S1. Relative gene expression levels were calculated using the 2^−△△Ct^ method. Three technical replicates per sample were averaged before analysis, and the three independent biological replicates were used as the statistical unit. Data are presented as the mean ± standard deviation (SD). Statistical analysis was performed with GraphPad Prism 9.5 (GraphPad Software, San Diego, CA, USA) using two-way ANOVA with genotype and treatment time as factors, followed by multiple-comparison tests, with significance levels indicated as * *p* < 0.05, ** *p* < 0.01, *** *p* < 0.001, and **** *p* < 0.0001, consistent with the figure legends.

### 4.6. Subcellular Localization of BhKCS Proteins

The coding sequences (CDS) of BhKCS8, BhKCS9, BhKCS14 and BhKCS15 were commercially synthesized (Beijing Tsingke Biotech Co., Ltd., Beijing, China) and cloned in-frame into the pR101-GFP vector, and the sequence of each construct was verified by Sanger sequencing before use. The resulting plasmids were introduced into Agrobacterium tumefaciens strain GV3101. Overnight cultures were grown at 28 °C, diluted 1:100 into fresh medium and grown overnight again, then harvested by centrifugation at 6000 rpm for 5 min at 4 °C. The cells were resuspended in infiltration buffer (10 mM MES, 10 mM MgCl_2_ and 150 µM acetosyringone, pH 5.6) and the suspension was adjusted to an OD_600_ of 0.6 and left to stand at room temperature for 1–3 h. Nicotiana benthamiana plants were grown in a growth chamber and healthy leaves of 2–4-week-old plants were used for infiltration. Each construct was infiltrated separately into independent leaf areas of separate plants; the four BhKCS constructs were not co-infiltrated within a single leaf. For plasma-membrane counterstaining, the 35S::PIP2-1-mCherry marker was co-infiltrated with each BhKCS-GFP construct, so that a single cell expressed only one BhKCS-GFP fusion together with the mCherry marker; no stable transgenic marker line was used. Infiltrated plants were kept under standard growth conditions and leaf samples were examined 48–72 h after infiltration. Fluorescence signals were observed and captured using a Zeiss LSM 900 confocal microscope (Carl Zeiss, Oberkochen, Germany).

### 4.7. Abiotic Stress Response Verification Using Yeast Overexpression System

To investigate the potential role of *BhKCS* genes in abiotic stress responses, yeast heterologous expression assays were performed. The coding sequences of *BhKCS8*, *BhKCS9*, *BhKCS14*, and *BhKCS15* were synthesized (Beijing Tsingke Biotech Co., Ltd., Beijing, China) and individually cloned into the pYES2 vector under the control of a galactose-inducible promoter. The recombinant plasmids, along with the empty pYES2 vector as a control, were transformed into the Saccharomyces cerevisiae strain INVSC1 (auxotrophic markers: his3, leu2, trp1, ura3) by the lithium acetate/PEG method, and the transformants were recovered in YPD Plus medium. Positive transformants were selected on synthetic defined (SD) medium lacking uracil (SD/-Ura) and incubated at 28 °C for 2–3 days.

For stress tolerance testing, single colonies harboring either the pYES2-BhKCS constructs or the empty vector were inoculated into liquid SG/-Ura induction medium containing 2% (*w*/*v*) galactose and cultured at 28 °C with shaking at 200 rpm for 3–5 days. Yeast cultures were then harvested, and the optical density at 600 nm (OD_600_) was adjusted to 1.0 with sterile water. Subsequently, 4 µL of serial ten-fold dilutions of each culture were spotted onto SG/-Ura solid medium supplemented with various stress agents: 5% (*w*/*v*) PEG6000, 300 mM NaCl, or medium adjusted to pH 4.0 with HCl, to simulate osmotic, salt, and acid stress conditions, respectively. The osmotic potential of each treatment was calculated using the empirical equation of Michel and Kaufmann [26], because the osmotic potential of PEG solutions varies non-linearly with concentration. Spot assays were performed in three independent experiments. Growth was scored as the lowest dilution at which visible colonies formed. The plates were incubated at 28 °C for 2–3 days. 

### 4.8. Scanning Electron Microscopy (SEM) Analysis

Mature wax gourd fruits of cultivars P and G were collected. The exocarp of fresh fruit was sliced into 5 mm sections and immediately fixed at room temperature for 2 h using an electron microscopy fixative (Code: G1102, Servicebio, Wuhan, China) for morphological observation. After fixation, the samples were stored and transported at 4 °C. Subsequently, they were fixed with 0.1 mol/L phosphate buffer (PB, pH 7.4), dehydrated through a graded ethanol series, and treated with isoamyl acetate. Following dehydration, the samples were dried, mounted on aluminum stubs with carbon adhesive tabs, and sputter-coated with a thin layer of gold using a Hitachi MC1000 coater (Hitachi, Tokyo, Japan) for 30 s. Finally, the samples were observed under a Hitachi SU8100 scanning electron microscope (Hitachi, Tokyo, Japan) operating at an accelerating voltage of 3.0 kV and a working distance of 10.2–15.2 mm, with the SE(L) signal and an acquisition resolution of 2560 × 1920 pixels. Magnification and scale-bar settings were recorded automatically by the microscope control software (PC-SEM, Hitachi High-Tech, Tokyo, Japan) for every image.

## 5. Conclusions

In this study, 27 *BhKCS* genes were systematically identified and characterized in wax gourd. The genes exhibited diverse phylogenetic relationships, structural features and promoter cis-element compositions. Functional assays in yeast showed that overexpression of BhKCS8 and BhKCS9 did not impair yeast growth under stress conditions, whereas overexpression of BhKCS14 and BhKCS15 inhibited yeast growth, suggesting potential but opposing effects of these genes in the yeast system. However, their actual regulatory functions in wax gourd remain to be investigated through in planta studies. The two types differ in cuticular wax microstructure and in stress-responsive gene expression, but direct physiological and wax-chemical evidence is required before a difference in stress tolerance can be claimed. These findings provide a foundation for further functional validation of *BhKCS* genes and identify candidate genes for molecular breeding of wax-related traits in wax gourd.

## Figures and Tables

**Table 1:** Information on the 27 BhKCS genes identified in the wax gourd genome. Subcellular localization was predicted using Plant-mPLoc.

Gene ID	Name	At	At-Name	Length	MW (Da)	pI	Predicted Localization
Bh.pf3chr1g537	BhKCS1	AT1G01120.1	KCS1	528	58,739.4	8.19	Cytoplasm
Bh.pf3chr1g778	BhKCS2	AT1G04220.1	KCS2	502	56,848.8	9.59	Cytoplasm
Bh.pf3chr5g1421	BhKCS3	AT1G19440.1	KCS4	454	50,979.8	8.26	Cytoplasm
Bh.pf3chr4g264	BhKCS4	AT1G19440.1	KCS4	517	57,745.4	9.26	Chloroplast Cytoplasm
Bh.pf3chr1g1378	BhKCS5	AT1G19440.1	KCS4	210	23,702.2	9.85	Cytoplasm
Bh.pf3chr5g1447	BhKCS6	AT1G19440.1	KCS4	423	47,315.6	8.26	Cytoplasm
Bh.pf3chr1g1243	BhKCS7	AT1G25450.1	KCS5	381	42,627.9	8.21	Cytoplasm
Bh.pf3chr7g1546	BhKCS8	AT1G68530.1	KCS6	496	55,968.1	9.46	Cytoplasm
Bh.pf3chr1g1380	BhKCS9	AT1G68530.1	KCS6	473	53,370.4	8.53	Cytoplasm
Bh.pf3chr12g491	BhKCS10	AT1G71160.1	KCS7	404	42,348.4	7.63	Chloroplast
Bh.pf3chr11g1805	BhKCS11	AT1G71160.1	KCS7	461	51,611.1	9.6	Cytoplasm
Bh.pf3chr9g3262	BhKCS12	AT2G26250.1	KCS10	551	63,054.1	9.28	Chloroplast Cytoplasm
Bh.pf3chr2g1142	BhKCS13	AT2G26640.1	KCS11	513	57,793.9	9.72	Cytoplasm
Bh.pf3chr8g708	BhKCS14	AT2G28630.1	KCS12	462	52,137.9	9.29	Cytoplasm
Bh.pf3chr10g605	BhKCS15	AT2G28630.1	KCS12	461	52,334.9	8.3	Cytoplasm
Bh.pf3chr1g1963	BhKCS16	AT3G52160.1	KCS15	400	44,158.5	5.94	Cytoplasm
Bh.pf3chr12g1709	BhKCS17	AT4G34250.1	KCS16	390	42,761.9	6.41	Cytoplasm
Bh.pf3chr1g565	BhKCS18	AT4G34250.1	KCS16	389	43,118.3	6.52	Cytoplasm
Bh.pf3chr5g1905	BhKCS19	AT4G34250.1	KCS16	395	43,237.7	6.19	Cytoplasm
Bh.pf3chr1g1823	BhKCS20	AT4G34520.1	KCS18	400	43,895.2	6.14	Cytoplasm
Bh.pf3chr7g1233	BhKCS21	AT5G04530.1	KCS19	241	27,106.3	8.71	Cytoplasm
Bh.pf3chr7g1230	BhKCS22	AT5G04530.1	KCS19	469	52,826.1	9	Cytoplasm
Bh.pf3chr7g1232	BhKCS23	AT5G04530.1	KCS19	191	21,851.5	7.98	Cytoplasm Nucleus
Bh.pf3chr1g42	BhKCS24	no		462	48,473.1	8.73	Peroxisome
Bh.pf3chr12g1710	BhKCS25	no		461	48,605.4	8.1	Peroxisome
Bh.pf3chr11g500	BhKCS26	no		472	52,317.9	6.03	Cytoplasm
Bh.pf3chr5g108	BhKCS27	no		453	47,578.1	7.79	Peroxisome

## Data Availability

The wax gourd genome data were downloaded from National Genomics Data Center (NGDC, https://ngdc.cncb.ac.cn/gwh/) (accession number: GWHBJVO00000000) or National Center for Biotechnology Information (NCBI, https://www.ncbi.nlm.nih.gov/) (accession number: GCA_027475165.1).

## Supplementary Materials

The following supporting information can be downloaded at https://www.mdpi.com/article/10.3390/plants15193076/s1: Supplementary Table S1. The primers list.

## Abbreviations

ACP	acyl carrier protein
BLASTP	Basic Local Alignment Search Tool for proteins
CDD	Conserved Domain Database
CDS	coding sequence
CER6	ECERIFERUM 6
FAE1	FATTY ACID ELONGATION 1
FDH	FIDDLEHEAD
HMM	Hidden Markov Model
KCS	β-ketoacyl-CoA synthase
MEME	Multiple Expectation Maximization for Motif Elicitation
NCBI	National Center for Biotechnology Information
NGDC	National Genomics Data Center
PB	phosphate buffer
PEG	polyethylene glycol
qRT-PCR	quantitative real-time polymerase chain reaction
SD	synthetic defined
SEM	scanning electron microscopy
VLCFA	very-long-chain fatty acid
